# Expert-Performed Endotracheal Intubation-Related Complications in Trauma Patients: Incidence, Possible Risk Factors, and Outcomes in the Prehospital Setting and Emergency Department

**DOI:** 10.1155/2018/5649476

**Published:** 2018-06-10

**Authors:** Yuko Ono, Takeyasu Kakamu, Hiroaki Kikuchi, Yusuke Mori, Yui Watanabe, Kazuaki Shinohara

**Affiliations:** ^1^Department of Anesthesiology, Ohta General Hospital Foundation, Ohta Nishinouchi Hospital, 2-5-20 Nishinouchi, Koriyama City, Fukushima 963-8558, Japan; ^2^Emergency and Critical Care Medical Center, Fukushima Medical University Hospital, 1 Hikarigaoka, Fukushima City, Fukushima 960-1295, Japan; ^3^Department of Pharmacology, School of Medicine, Fukushima Medical University, 1 Hikarigaoka, Fukushima City, Fukushima 960-1295, Japan; ^4^Department of Hygiene and Preventive Medicine, School of Medicine, Fukushima Medical University, 1 Hikarigaoka, Fukushima City, Fukushima 960-1295, Japan

## Abstract

The aim of this study was to determine complication rates and possible risk factors of expert-performed endotracheal intubation (ETI) in patients with trauma, in both the prehospital setting and the emergency department. We also investigated how the occurrence of ETI-related complications affected the survival of trauma patients. This single-center retrospective observational study included all injured patients who underwent anesthesiologist-performed ETI from 2007 to 2017. ETI-related complications were defined as hypoxemia, unrecognized esophageal intubation, regurgitation, cardiac arrest, ETI failure rescued by emergency surgical airway, dental trauma, cuff leak, and mainstem bronchus intubation. Of the 537 patients included, 23.5% experienced at least one complication. Multivariable logistic regression analysis revealed that low Glasgow Coma Scale Score (adjusted odds ratio [AOR], 0.93; 95% confidence interval [CI], 0.88–0.98), elevated heart rate (AOR, 1.01; 95% CI, 1.00–1.02), and three or more ETI attempts (AOR, 15.71; 95% CI, 3.37–73.2) were independent predictors of ETI-related complications. We also found that ETI-related complications decreased the likelihood of survival of trauma patients (AOR, 0.60; 95% CI, 0.38–0.95), independently of age, male sex, Injury Severity Score, Glasgow Coma Scale Score, and off-hours presentation. Our results suggest that airway management in trauma patients carries a very high risk; this finding has implications for the practice of airway management in injured patients.

## 1. Introduction

Traumatic injury is the leading cause of death and disability among young people and places a tremendous economic burden on society [1, 2]. Early and appropriate airway management is a vital lifesaving measure in severely injured patients [3] because airway compromise is a significant cause of poor outcomes in this population [4]. Trauma patients have a very high risk of hemodynamic instability, restlessness, inadequate evaluation, and the need for cervical spine protection, all of which contribute to difficult ETI [5–9]. Ideally, this challenging procedure should be delegated to a skilled laryngoscopist who performs ETI frequently, such as an anesthesiologist. To better understand the risks of trauma airway management, it is important to clarify the incidence and associated factors of expert-performed ETI-related complications. However, past studies have not fully provided this information.

Previous studies have reported that trauma ETI can cause life-threating complications, including aspiration, misplacement of the endotracheal tube, hypoxemia, the need for an emergency surgical airway, and circulatory collapse [8, 9]. However, the degree to which these complications affect the outcomes of trauma patients is not well understood.

In our hospital emergency department (ED), airway management in severe trauma patients is primarily the responsibility of anesthesiologists. Our facility also runs a prehospital emergency medical unit staffed by the same anesthesiologists. This unique clinical arrangement allows us to investigate the incidence and possible risk factors of ETI-related complications in trauma patients treated in both the prehospital setting and the ED by laryngoscopists with “expert” status, as recently defined by Breckwoldt et al. [10]. Using our trauma and quality assurance database, we also investigated how the occurrence of ETI-related complications affected the survival of trauma patients.

## 2. Materials and Methods

### 2.1. Ethical Approval and Consent to Participate

The study was approved by the Ethics Committee of Ohta Nishinouchi Hospital (no. 6_H27) on May 15, 2015. The committee waived the need for informed consent because the study was nonrandomized and assessed the clinical outcomes of routine practices.

### 2.2. Study Design and Setting

This retrospective observational study was conducted at a community hospital in a provincial Japanese city located approximately 200 km north of Tokyo. The hospital serves both as a teaching facility and as a referral medical center for a population of 538,000 inhabitants within an area of 2400 km^2^. Annually, the hospital receives > 5500 ambulances and > 1200 trauma patients with injuries of varying severity. At the hospital, the primary responsibility for airway management in severe trauma patients lies with ED physicians with a background in anesthesiology. The hospital also runs a prehospital emergency medical unit (doctor-car system) consisting of a trained ambulance driver, a nurse, and a senior physician whose specialty is anesthesiology. This physician-delivery system is dispatched to the scene in response to a request by the regional medical control center.

Anesthesiologists must have a minimum of 4 years of clinical experience before they are allowed to work in the ED or the prehospital emergency medical unit. Each anesthesiologist performs approximately 300 ETIs per year in the operating room. Operating room cases cover the full spectrum of difficult airway situations, including head and neck surgery, pediatric anesthesia, and differential lung ventilation.

### 2.3. Participants and Data Sources

This study included all trauma patients who underwent emergency ETI in either the prehospital or the ED setting from January 1, 2007, to January 1, 2017. The exclusion criteria were as follows: patients who received ongoing cardiopulmonary resuscitation at initial contact, patients who received a surgical airway as an initial intubation attempt, and patients who were transported to our hospital from other facilities. In Japan, paramedics are not permitted to perform ETI except in cases of cardiopulmonary arrest. Therefore, this study did not include trauma patients who received ETI performed by paramedics. The data were collected from a trauma and quality assurance electronic database, medical records, and nursing records. Our facility maintains a rigorous peer-review process to ensure the quality of our trauma practice. Life-threatening ETI-associated complications, such as cardiac arrest after an ETI attempt, failed intubation salvaged by emergency surgical airway, and esophageal intubation with delayed recognition, occurring in either the prehospital or ED setting, are peer-reviewed, confirmed by experienced anesthesiologists, and recorded in the quality assurance database.

The database also records injury severity according to the Abbreviated Injury Scale (AIS) for each body region [11], the Injury Severity Score (ISS) [12], the Revised Trauma Score (RTS) [13], and the probability of survival (Ps), which is based on the Trauma and Injury Severity Scores method [14, 15]. These parameters were scored without delay by one of the authors (KS). Comorbidity was described using the Charlson comorbidity index [16].

Our department uses a structured medical record that includes the patient's age, sex, initial vital signs, time from the emergency call to scene arrival, time from scene departure to the ED, past medical history, detailed history of the present condition, physical examination, laboratory data, radiological findings, final diagnosis, patient disposition, and any complications. As specified in the guidelines of several professional anesthesiology societies [17, 18], our department mandates documentation of the details of airway difficulty in the medical record to provide relevant information on ETI-related complications. All physicians who participate in the management of trauma patients are required to complete the form immediately; an ED director at our hospital (KS) checks all medical records at the earliest possible opportunity to verify the completeness and reliability of the data. Nursing records include information on the laryngoscopist, the number of ETI attempts, the medication used to facilitate ETI, and the patient's vital signs before and after ETI attempts. The ETI procedure and the choice of drugs to facilitate ETI are at the discretion of the participating anesthesiologist. A standard operating procedure for ETI [19], such as unified equipment set-up, pre-ETI assessment, and postintubation care with end-tidal CO_2_ detection, has not yet been established in our facility. Correct endotracheal tube placement is verified based on clinical findings, such as tube fogging, chest rise, and auscultation, with secondary confirmation by capnometry performed at the discretion of the attending physician. In our ED, a chest X-ray or computed tomography scan is routinely performed after tube placement to detect mainstem bronchus intubation. For consistency with our own studies and those of other researchers [20, 21], off hours were defined as the period from 6:01 PM to 8:00 AM on weekdays plus the entire weekend.

### 2.4. Definition of Endotracheal Intubation-Related Complications

ETI-associated complications were defined as hypoxemia, esophageal intubation with delayed recognition, cardiac arrest immediately after ETI attempt, recorded regurgitation, ETI failure rescued by emergency surgical airway, dental trauma, cuff leak requiring intubation, or mainstem bronchus intubation. Hypoxemia was defined as a decline in pulse oximetry saturation > 10% from baseline during ETI attempts, not resulting from esophageal intubation [21–24]. Esophageal intubation with delayed recognition was defined as misplacement of the endotracheal tube in the upper esophagus or hypopharynx, with time elapsed and desaturation (> 10% decline in pulse oximetry saturation) also recorded [21–24]. Recorded regurgitation was defined as the immediate peri-induction regurgitation of gastric contents at the glottis opening or in the endotracheal tube, clearly documented in the ED or nursing records [21–24]. Cardiac arrest immediately after an ETI attempt included asystole, bradycardia, or dysrhythmia in a patient without a measurable blood pressure and requiring cardiopulmonary resuscitation during or immediately after the ETI attempt [21–24]. If ETI was impossible after anesthesia induction and a salvage surgical technique was required, then the event was classified as ETI failure rescued by emergency surgical airway [21, 25]. Previous studies have included hemodynamic parameters, such as hypertension and hypotension, in the definition of ETI-related complications [22–24]. However, we chose to exclude hemodynamic data because hemodynamic perturbations resulting from ETI are difficult to distinguish from those arising from underlying trauma-based etiologies [26, 27].

### 2.5. Objectives of the Study

The main objective of the study was to clarify the incidence and possible risk factors of ETI-related complications in patients with trauma. The secondary aim was to assess how ETI-related complications affect the survival of trauma patients.

### 2.6. Statistical Analysis

To achieve the study goals, we first evaluated differences in the baseline clinical characteristics of trauma patients who experienced ETI-related complications versus those who did not. Differences in continuous variables were compared with Student's* t*-test or Mann–Whitney* U*-test for normally and non-normally distributed data, respectively, after applying the Shapiro–Wilk test for normality. Differences in categorical variables were compared with a chi-squared test followed by residual analysis.

Next, univariable and multivariable logistic regression models were established to detect independent risk factors for ETI-related complications. Imbalanced characteristics between patients with versus without ETI-related complications (variables with P < 0.15 in Table 2; see Results), such as the location in which ETI was performed, use of rapid-sequence intubation technique, patient sex, ISS, GCS, heart rate, pulse oximetry saturation, and need for three or more ETI attempts, were included as independent variables in the logistic regressions.

Finally, to clarify whether ETI-related complications decreased the survival of injured patients independently of age, sex, ISS, GCS, and off-hours admission, additional logistic regression models were constructed. A set of these variables was chosen a priori based on previous information [12, 28–30] and biological plausibility.

In all logistic regression models, a variance inflation factor was used to detect multicollinearity. The models' goodness of fit and discrimination ability were confirmed with the Hosmer–Lemeshow test and the* c* statistic, respectively. All statistical analyses were performed with SPSS Statistics for Windows, version 22.0 (IBM Corp., Armonk, NY, USA). A P value < 0.05 was considered statistically significant.

## 3. Results

**Figure 1:**
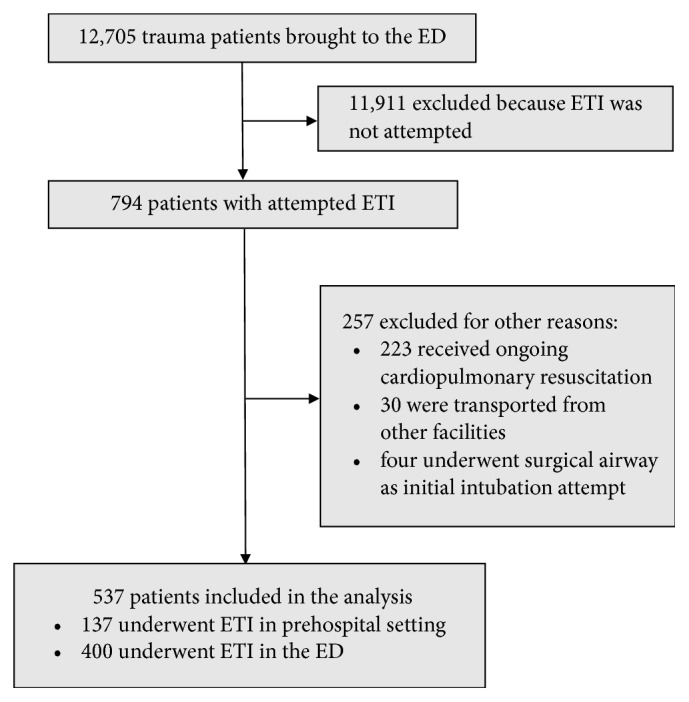
**Flow chart showing the selection process for injured patients who underwent ETI in prehospital or emergency department setting.** *ED *emergency department,* ETI *endotracheal intubation.

During the 120-month study period, 12,705 trauma patients were brought to the ED, of whom 794 required ETI in the prehospital or ED setting (Figure 1). Of these, we excluded 223 patients who received ongoing cardiopulmonary resuscitation, 30 patients who were transported from other facilities, and four patients who underwent initial airway management by emergency surgery. The remaining 537 patients were included in the analysis. Of these patients, 137 (25.5%) received ETI in a prehospital setting and 400 (74.5%) in the ED (Figure 1). Complete records were available for all patients and no data were missing from the analyses.

### 3.1. Incidence of Endotracheal Intubation-Related Complications

Table 1 shows details of ETI-related airway complications in prehospital and ED settings. Overall, 23.5% of the study population experienced at least one ETI-associated complication, of which mainstem bronchus intubation was the most common.

### 3.2. Possible Risk Factors for Endotracheal Intubation-Related Complications

Table 2 compares the clinical demographics of patients who experienced ETI-related complications versus those who did not. Patients who experienced ETI-related complications were more likely to have higher scores for anatomic severity scales (ISS, P = 0.005; AIS head or neck, P = 0.036), lower scores for physiological severity scales (GCS, P < 0.001; RTS, P < 0.001), and higher heart rate (P = 0.011) than those without complications. The occurrence of ETI-related complications was significantly higher in the prehospital setting (crude OR, 1.75; 95% CI, 1.13–2.70; P = 0.011) and in patients who needed three or more ETI attempts (crude OR, 21.53; 95% CI, 4.75–97.6; P < 0.001). Rapid-sequence intubation technique was used in 34.9% of patients who experienced airway complications and in 51.1% of patients who did not.

Imbalanced characteristics (variables in Table 2 with P < 0.15) were entered into a multivariable model, which revealed that the independent predictors of ETI-related complications in injured patients were GCS (adjusted odds ratio [AOR], 0.93; 95% confidence interval [CI], 0.88–0.98; P = 0.009), heart rate (AOR, 1.01; 95% CI, 1.00–1.02; P = 0.023), and three or more ETI attempts (AOR, 15.71; 95% CI, 3.37–73.2; P < 0.001) (Table 3). The Hosmer–Lemeshow test verified the good fit of this model (P = 0.139); the c statistic for this logistic model was 0.704, suggesting acceptable discrimination. The use of rapid-sequence intubation technique was associated with a lower risk of airway-related complications on crude analysis (crude OR, 0.51; 95% CI, 0.34–0.78; P = 0.002). A similar association remained on adjusted analysis, although it did not reach statistical significance (AOR, 0.67; 95% CI, 0.41–1.08; P = 0.099).

### 3.3. Endotracheal Intubation-Related Complications Independently Worsened the Survival of Trauma Patients

A second multivariable logistic regression analysis showed that ETI-related complications decreased the likelihood of survival of trauma patients (AOR, 0.60; 95% CI, 0.38–0.95; P = 0.030), independently of age, male sex, ISS, GCS, and off-hours presentation (Table 4). The Hosmer–Lemeshow test verified the good fit of this model (P = 0.161); the c statistic for this logistic model was 0.784, suggesting acceptable discrimination.

## 4. Discussion

In this study of trauma patients who underwent expert-performed ETI for airway compromise, severe complications were common and were associated with low GCS, elevated heart rate, and the need for three or more ETI attempts. We also found that ETI-related complications decreased the likelihood of survival of trauma patients, independently of age, sex, anatomic severity, and physiological severity.

Our data showed that even laryngoscopists with expert status [10] may frequently be confronted with severe ETI-related complications in both the prehospital and ED settings. Our results suggest that trauma airway management carries a very high risk; therefore, our findings serve as a caution to healthcare professionals involved in this procedure. Although this study did not directly address this issue, we believe that the experience of the laryngoscopist should be considered when performing ETI in injured patients. Previous studies have indicated that prehospital ETI performed by paramedics with limited experience results in twice the rate of esophageal intubation as ETI performed by experienced physicians [31–34]. Reported overall prehospital ETI success rates in patients with trauma are 68% for paramedics [31] and 99.3% for trauma anesthesiologists [9]. This ETI success rate for anesthesiologists was comparable with our data (98.7% success rate). A previous study [35] and a recent meta-analysis [36] also showed that the provider's degree of ETI experience significantly influenced the outcomes of injured patients. In addition, Paal et al. [37] and the Scandinavian Society for Anaesthesiology and Intensive Care Medicine [38] recommend that ETI in high risk populations should be delegated to skilled laryngoscopists.

We also found that low GCS, elevated heart rate, and multiple ETI attempts were potential risk factors for ETI-related complications. Higher heart rate and lower GCS may reflect the lower physiological reserve of injured patients, which predisposes them to airway-related complications. Our findings highlight the need for care providers to be especially vigilant in treating such patients.

Consistent with previous reports [23, 24, 27], repeated attempts at laryngoscopy were associated with ETI-related complications in our trauma cohort. This finding supports the use of strategies to limit the number of laryngoscopy attempts and maximize first-pass laryngoscopy success when performing ETI, such as optimal positioning and use of airway management adjuncts (e.g., gum elastic bougie or video laryngoscopy) [23].

Crude analysis in the present study showed that the use of rapid-sequence intubation technique was associated with a lower risk of airway-related complications in injured patients. A similar association remained after adjustment for injury and physiological severity, although it did not reach statistical significance. Previous studies have documented the associations between the use of rapid-sequence intubation and high ETI success rates [22, 39–41] and low complication rates [42]. Our data corroborate these findings and expand them to include a different patient population and practice setting. Along with the existing literature, our findings support the current practice guideline [43, 44], which recommends rapid-sequence intubation as the initial method of emergency airway management in most trauma patients.

Finally, we found that ETI-related complications decreased the likelihood of survival of trauma patients, independently of anatomic severity and physiological reserve, in both the prehospital setting and the ED. Therefore, all healthcare professionals should be aware that any airway-related complication increases the risk of further harm in trauma patients. Although this study was unable to confirm the hypothesis, it is possible that a considerable proportion of observed complications might have been avoided. Jaber et al. [45] recently reported that the introduction of an “intubation bundle,” including preoxygenation, rapid-sequence intubation, and capnography to verify correct tube placement, significantly decreased the number of ETI-related complications in critically ill patients. In many Japanese EDs, including our own, procedural preferences for ETI vary greatly [46] and a standardized procedure is lacking. To reduce ETI-related complications, a standard operating procedure [19] for ETI in trauma patients should be implemented (e.g., unified equipment set-up, rapid-sequence intubation, and postintubation care with end-tidal CO_2_ detection) in both the prehospital setting and ED. We believe that implementing standardized procedures will improve the outcomes of trauma patients.

### 4.1. Limitations and Strengths

This study had three major limitations. First, its retrospective nature may have increased the risk of bias, including self-reporting and diagnostic biases. Despite the use of structured medical records and a quality assurance database that captured all severe ETI-related complications occurring in both prehospital and ED settings, ETI complications may have been missed, underestimated, or misclassified.

Second, although multivariable logistic regression analysis indicated that GCS, heart rate, and the need for three or more ETI attempts were potential risk factors for ETI-related complications, there may have been other, unknown confounders for ETI-related complications, as can occur in any observational study. For example, board certification in anesthesiology of the operators and the use of a video laryngoscope [47, 48] or capnometry [49] may have affected the rate of ETI success and ETI-related complications. However, our database did not record these variables. There were also substantial differences in characteristics between patients who experienced airway-related complications and those who did not. Although we rigorously adjusted for these differences to detect independent predictors of airway-related complications, there remains a risk of incomplete adjustment. For example, ISS has been shown to underestimate multiple severe injuries within the same body region [50]. It might have been useful to use the new ISS (the sum of squares of the three most severe injuries, regardless of the body regions injured) [51] instead of the ISS; however, we did not record this variable.

Third, while our ED is typical of a Japanese teaching hospital, as with any single-center study, it may not be possible to extrapolate our findings to other medical institutions, especially those in other countries.

Despite these limitations, this study also had several strengths. First, our study clarified the incidence and risk factors for ETI-related complications in trauma patients when expert laryngoscopists performed ETI in the ED and prehospital settings. Our hospital's anesthesiologists have long been in charge of trauma airway management in both locations. To the best of our knowledge, past studies have not provided such information. Second, because we used structured ED records and our department has a rigorous peer-review process supervised by its director, there were no missing data. We therefore believe that our study provides an accurate depiction of advanced airway management by expert laryngoscopists in trauma patients in prehospital and ED settings.

## 5. Conclusion

In this study of trauma patients who underwent expert-performed ETI for airway compromise, severe ETI-related adverse events were common and were associated with low GCS, elevated heart rate, and repeated ETI attempts. The occurrence of these airway-related complications decreased the likelihood of survival of injured patients, independently of anatomic severity and physiological reserve. These data have implications for the practice of airway management in trauma patients in the prehospital setting and ED.

## Figures and Tables

**Table 1:** Details of airway-related complications in injured patients who underwent ETI in prehospital or emergency department setting.

***N* = 537**	**n (%)**
At least one complication	126 (23.5)
Hypoxemia	25 (4.7)
Esophageal intubation with delayed recognition	9 (1.7)
Cardiac arrest immediately after ETI attempt	17 (3.2)
Recorded regurgitation	27 (5.0)
ETI failure rescued by emergency surgical airway	7 (1.3)
Dental trauma	8 (1.5)
Cuff leak requiring reintubation	5 (0.9)
Mainstem bronchus intubation	46 (8.6)

Data are expressed as n (%). *ETI:* endotracheal intubation.

**Table 2:** Demographic characteristics of trauma patients, according to occurrence of ETI-related complications.

	**Number of ETI-related complications**		
	**≥ 1 (*n *=126)**	**0 (*n *=411)**	**P value**
Age, years	59 (32–74)	60 (37–73)	0.864
Male, *n* (%)	80 (63.5)	291 (70.8)	0.121
Etiology of trauma, *n* (%)			0.499
Blunt injury	102 (81.0)	327 (79.6)	
Penetrating injury	7 (5.6)	25 (6.1)	
Burn	17 (13.5)	59 (14.4)	
Anatomic parameters			
AIS			
Head or neck	3 (0–5)	2 (0–5)	0.036
Face	0 (0–1)	0 (0–1)	0.288
Chest	3 (0–4)	3 (0–4)	0.665
Abdomen or pelvic contents	0 (0–2)	0 (0–2)	0.526
Extremities or pelvic girdle	2 (0–3)	1 (0–3)	0.870
External	0 (0–0)	0 (0–0)	0.679
ISS	36 (25–45)	29 (22–42)	0.005
Physiological parameters			
GCS	8 (3–12)	12 (6–14)	< 0.001
Initial recorded vital signs			
Systolic blood pressure, mmHg (mean [SD])	126 (41)	125 (40)	0.916
Heart rate, beats/min	101 (85–120)	92 (78–116)	0.011
Shock index	0.8 (0.6–1.1)	0.8 (0.6–1.0)	0.267
Respiratory rate, breaths/min	24 (20–30)	22 (18–30)	0.072
Pulse oximetry saturation, %	100 (95–100)	100 (98–100)	0.128
RTS	5.9 (4.2–6.9)	6.5 (5.1–7.8)	< 0.001
Ps, %	59.0 (18.8–85.4)	80.9 (40.4–95.0)	< 0.001
Charlson comorbidity index	0 (0–1)	0 (0–1)	0.579
Off-hours presentation^a^, *n* (%)	76 (60.3)	246 (59.9)	0.926
Emergency operation,* n* (%)	54 (42.9)	204 (49.6)	0.183
Prehospital ETI	43 (34.1)	94 (22.9)	0.011
Prehospital times			
From emergency call to scene arrival, min	9 (7–13)	10 (7–13)	0.877
From scene arrival to departure, min	12 (7–19)	12 (7–21)	0.96
From scene departure to hospital arrival, min	12 (8–22)	14 (8–24)	0.473
Total prehospital time, min	37 (27–57)	38 (27–59)	0.652
Characteristics of ETI			
Three or more ETI attempts	12 (9.5)	2 (0.5)	< 0.001
ETI method			0.005
Without medication	45 (35.7)*∗*	90 (21.9)*∗∗*	
Sedative/analgesic only	28 (22.2)	80 (19.5)	
Paralytic agent only	9 (7.1)	31 (7.5)	
Rapid sequence intubation technique	44 (34.9)*∗∗*	210 (51.1)*∗*	
Hospital length of stay, days	42 (12–63)	43 (19–79)	0.550

Data are expressed as the median (interquartile range) unless otherwise indicated.

*AIS:* Abbreviated Injury Scale, *ED:* emergency department, *ETI:* endotracheal intubation, *GCS:* Glasgow Coma Scale Score, *ISS:* Injury Severity Score, *Ps: *probability of survival, *RTS:* Revised Trauma Score, *SD:* standard deviation.

^a^6:01 PM to 8:00 AM on weekdays plus all weekend hours.

*∗* Adjusted standardized residual > 1.96, *∗∗* adjusted standardized residual < −1.96.

**Table 3:** Logistic regression models for occurrence of airway-related complications in injured patients.

	**Univariable analysis **		**Multivariable analysis ** ^**a**^	
	**Crude OR (95% CI)**	**P value**	**AOR (95% CI)**	**P value**
Prehospital ETI	1.75 (1.13–2.70)	0.011	0.99 (0.58–1.66)	0.955
Male	0.72 (0.47–1.09)	0.121	0.64 (0.41–1.01)	0.053
Rapid-sequence intubation	0.51 (0.34–0.78)	0.002	0.67 (0.41–1.08)	0.099
AIS head or neck	1.10 (1.00–1.21)	0.041	-	-
ISS	1.02 (1.01–1.03)	0.002	1.01 (1.00–1.02)	0.161
GCS	0.90 (0.86–0.95)	< 0.001	0.93 (0.88–0.98)	0.009
RTS	0.82 (0.73–0.92)	0.001	-	-
Ps	0.33 (0.18–0.58)	< 0.001	-	-
Heart rate	1.01 (1.00–1.02)	0.022	1.01 (1.00–1.02)	0.023
Respiratory rate	1.01 (0.99–1.03)	0.230	1.01 (0.99–1.04)	0.203
Three or more ETI attempts	21.53 (4.75–97.6)	< 0.001	15.71 (3.37–73.2)	< 0.001
Pulse oximetry saturation	1.00 (0.99–1.00)	0.389	1.00 (0.99–1.01)	0.733

*AIS:* Abbreviated Injury Scale, *AOR: *adjusted odds ratio,* CI:* confidence interval, *ED:* emergency department, *ETI:* endotracheal intubation, *GCS:* Glasgow Coma Scale Score, *ISS:* Injury Severity Score, *OR:* odds ratio,* Ps: *probability of survival, *RTS:* Revised Trauma Score.

^a^Adjustment for all variables included in the table. The patient group that did not experience ETI-related complication was the reference set.

Good fit was verified with the Hosmer–Lemeshow test (P = 0.139). The *c *statistic for the model was 0.704. “AIS head or neck” was not used as an explanatory variable because of its strong correlation with GCS. RTS is a weighted physiological scoring system consisting of the GCS, systolic blood pressure, and respiratory rate. Therefore, RTS was not included as an explanatory variable in this model. Ps was not included because it is calculated from ISS, RTS, and age.

**Table 4:** Logistic regression models of factors associated with survival in trauma patients who received ETI in the prehospital setting or ED.

	**Univariable analysis **			**Multivariable analysis** ^**a**^	
	**Crude OR (95% CI)**	**P value**		**AOR (95% CI)**	**P value**
ETI-related adverse events	0.46 (0.31–0.69)	< 0.001		0.60 (0.38–0.95)	0.030
Age	0.98 (0.97–0.99)	< 0.001		0.98 (0.97–0.98)	< 0.001
Male	1.06 (0.73–1.55)	0.744		0.95 (0.61–1.47)	0.814
ISS	0.95 (0.94–0.97)	< 0.001		0.96 (0.95–0.97)	< 0.001
GCS	1.18 (1.13–1.23)	< 0.001		1.16 (1.10–1.21)	< 0.001
Off-hours^b^ presentation	0.87 (0.61–1.24)	0.439		0.74 (0.49–1.13)	0.160

ETI-related complications independently worsened the survival of trauma patients.

*AOR: *adjusted odds ratio, *CI:* confidence interval, *ED:* emergency department, *ETI:* endotracheal intubation, *GCS:* Glasgow Coma Scale Score, *ISS:* Injury Severity Score, *OR:* odds ratio.

^a^Adjustment for all variables included in the table. Good fit was verified with the Hosmer–Lemeshow test (P = 0.161). The *c *statistic for the model was 0.784.

^b^6:01 PM to 8:00 AM on weekdays plus all weekend hours.

## Data Availability

All data relevant to the study are included in this published article. Further datasets analyzed during the study are available from the corresponding author on reasonable request.
